# The Effect of Detethering Surgery on the Bladder Function and Psychology of Children with Primary Tethered Cord Syndrome

**DOI:** 10.1590/S1677-5538.IBJU.2024.0311

**Published:** 2025-01-10

**Authors:** Shuai Yang, Zhaokai Zhou, Xingchen Liu, Zhan Wang, Yanping Zhang, He Zhang, Lei Lv, Yibo Wen, Qingwei Wang, Wei Jia, Jinhua Hu, Jian Guo Wen

**Affiliations:** 1 The First Affiliated Hospital of Zhengzhou University Paediatric Urodynamic Centre and Department of Urology Henan China Paediatric Urodynamic Centre and Department of Urology, The First Affiliated Hospital of Zhengzhou University, Henan 450052, China; 2 Henan Joint International Paediatric Urodynamic Laboratory Henan China Henan Joint International Paediatric Urodynamic Laboratory, Henan 450052, China; 3 Xinyang Central Hospital Department of Gynecology Henan Henan China Department of Gynecology, Xinyang Central Hospital, Xinyang 464000, Henan, China; 4 Guangzhou Women and Children's Medical Center Guangzhou China Guangzhou Women and Children's Medical Center, Guangzhou 510000, China

**Keywords:** Neural Tube Defects, Urodynamics, Psychology, Urinary Bladder, Neurogenic

## Abstract

**Purpose:**

Currently, detethering surgery (DS) is the modality most extensively utilized to treat primary tethered cord syndrome (TCS). Disappointingly, some children without bladder impairment showed a deterioration of bladder function after surgery, which critically influences the health-related quality of life. It was hypothesized that the DS might have a significant effect on bladder function and psychology. Therefore, the purpose of this study was to investigate the effect of DS on bladder function and quality of life in children with primary TCS.

**Materials and Methods:**

A retrospective study of 83 patients aged 6 to 10 years who were diagnosed with TCS and underwent DS between 2022 and 2023. The urodynamic parameters and score, psychological-behavioral profile, and lower urinary tract symptoms were compared before and after DS. Additionally, the patients were divided into the normal group and the abnormal group according to the preoperative urodynamics parameters.

**Results:**

A total of 66 children fulfilled the criteria, with a mean age at surgery of 89.5 ± 13.7 months. There were statistically significant differences in bladder detrusor sphincter synergy and urodynamic score and no statistically significant difference in the remaining urodynamic parameters and psychological-behavioral items before and after DS. The proportion of bladder dysfunction that improved or did not worsen after surgery was higher in the Abnormal group than in the Normal group. Nevertheless, the detection rate of each psychological behavior abnormality in children with TCS was higher compared with that of normal children, both preoperatively and postoperatively.

**Conclusions:**

DS could not considerably ameliorate pre-existing bladder dysfunction and patients exhibiting non-progressive bladder dysfunction could be treated conservatively with close observation. TCS plagues patients all the time even if detethering. Psychological counseling for children with TCS should be strengthened after DS.

## INTRODUCTION

Tethered cord syndrome (TCS) is presumed to be a diverse clinical entity characterized by the increased tension of the conus medullaris or cauda equina due to a comparatively low-lying level. Overstrain of the spinal cord could lead to "traction" ischemia and impaired oxidative metabolism, manifesting a constellation of clinical symptoms and signs of tension-induced cord dysfunction, predominantly back pain, orthopedic deformities, neurological deficits, and intestinal and urological disturbances (1). TCS is usually divided into primary and secondary TCS. The primary TCS is commonly seen in children and most of them show the different forms of congenital spinal closure disorders. The secondary form is an acquired pathology caused by previous operations, scar tissue development after open dysraphism closure, inflammation, or neoplasia (2, 3).

Primary TCS is one of the main causes of neurogenic bladder and its clinical manifestations include different types of low urinary tract symptoms (LUTS). Currently, DS is one of the invasive procedures to treat primary TCS but the results are controversial (4-7). Bowman and colleagues (5) reported that 64% of patients showed improvements in voiding function after DS. On the other hand, Valentini et al. (6) demonstrated that early DS was unable to halt preoperative urodynamic impairment, and radical surgery carried a high risk of new neuro-urological deterioration in chaotic lipoma and terminal myelocystocele. Additionally, some children suffering from primary TCS diagnosed by MRI, without LUTS, showed a bladder impairment after DS, which critically influenced the health-related quality of life (4). No doubt, current patients’ treatment selection is suboptimal, leading to over- or under-treatments. Obviously, further observation and research on the therapeutic effect of DS from the perspective of bladder function and quality of life is warranted.

It was hypothesized that a DS might have a significant effect on bladder function and psychology. In this study, children with primary TCS were investigated and followed up after DS. The therapeutic impact of surgery on bladder function was assessed by comparing urodynamic parameters pre- and post- detethering. Moreover, LUTS and psychological behavior modification in patients were quantitatively evaluated by questionnaires. Ultimately, we investigated the effect of DS on bladder function in children with primary TCS.

## MATERIAL AND METHODS

### Patient spectrum

**Figure 1:**
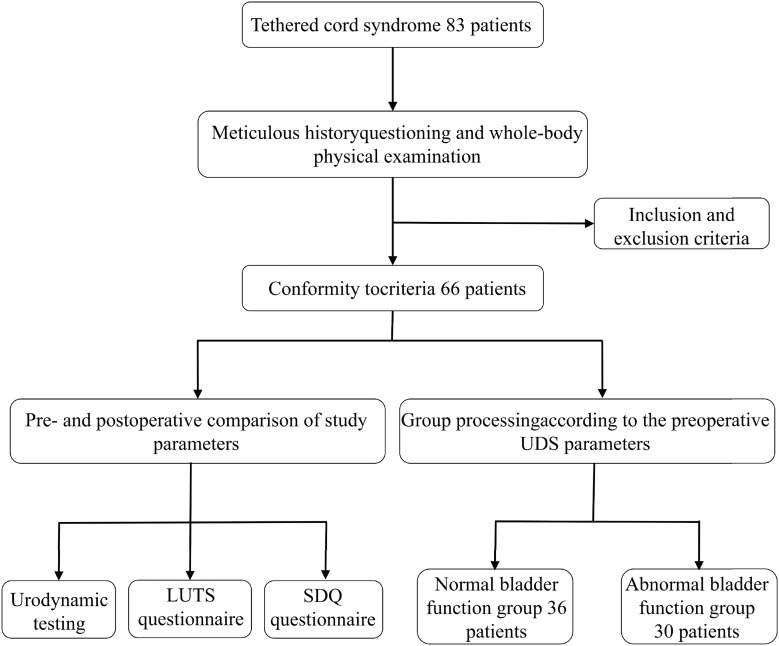
The flow chart of the study.

A total of 83 children aged 6 to 10 years with primary TCS consulted in the Pediatric Urodynamic Center of the First Affiliated Hospital of Zhengzhou University from October 2022 to December 2023 were retrospectively reviewed. This study was approved by the hospital Ethics Committee (IRB 2018-NY-86). Primary TCS was diagnosed in children after spinal MRI (fatty or thickened filum signals/conus medullaris located caudal to L2). At the time of the initial clinic visit, each case underwent meticulous history questioning and whole-body physical examination. Detailed historical information should pay attention to surgical history, medications, family history, voiding habits, and whether the patient ever experienced urologic symptoms. Physical examination includes the dorsal spine, cutaneous stigmata, and the lower extremity motor and sensory function. Sensory assessment should cover pinprick, tactility, and proprioception. Evaluation for defecation is also imperative. The flow chart is shown in Figure-1.

The patients were included after meeting the following criteria: [1] Children 6 to 10 years old diagnosed with primary TCS based on clinical symptoms, signs, and imaging (spinal cord cone located below the L2 vertebral body on MRI); [2] Underwent DS for the first time; [3] Urodynamic testing within 1 month prior to surgery (8); [4] Comprehensive case data and cooperated with follow-up for more than 3 months.

Exclusion criteria: [1] Underwent multiple DS; [2] Children with other significant comorbidities, such as myelitis, Guillain-Barre syndrome, and myasthenia gravis, shared similar symptoms with primary TCS and affected the determination of postoperative efficacy; [3] Diagnosed with primary TCS but with other diseases leading to urinary tract malformations, skeletal developmental malformations, and bowel malfunction; [4] Incomplete urodynamic parameters and case data before and after surgery and missing visits.

### Surgical procedure and conservative therapy

All patients underwent total resection of spinal lipomas by the same group of neurosurgeons. The patient was placed in the prone position, and intravenous inhalation anesthesia was routinely administered. The subcutaneous lipoma was separated up to the vertebral defect, and the vertebral plate was incised at the head of the defect to expose the normal anatomy, and then the subcutaneous lipoma was progressively removed from head to tail and into the spinal canal cavity. The spinal cord and cones were detached from the dura matter where they were attached by cutting with microscopic scissors to release the embolism. The end filaments were then explored and cut off if infiltrated with fat. Finally, the split spinal cord was closed with interrupted sutures, the dura was closed tightly, and the paraspinal muscles, lumbosacral fascia, subcutaneous fat, and skin were sutured.

Patients were selected for appropriate treatment options such as clean intermittent catheterization, anticholinergics, clean intermittent catheterization combined with anticholinergics, and behavioral therapy (pelvic floor muscle training, etc.) before and after surgery, according to the patient's urodynamic findings and symptoms (9, 10).

## STUDY PARAMETERS

### Urodynamic testing

Urodynamics (UDS) constitutes an indispensable modality for urologists to assess the function of lower urinary tract. Moreover, UDS is administered according to the methods recommended by the International Children's Continence Society (ICCS) (11). The UDS parameters included maximum flow rate (Qmax), post void residual (PVR), maximum cystometric bladder capacity (MCC), bladder compliance (BC), and detrusor activity (detrusor instability or hyperreflexia). In addition, the detrusor-sphincter synergy during voiding was assessed by recording the EMG activity of the external anal sphincter with perineal surface electrodes. Pre- and postoperative Qmax, PVR, MCC, BC, detrusor activity, detrusor-sphincter synergy, and UDS scores (Table-S1) were compared in children with primary TCS. Meyrat et al. introduced for the first time that the UDS score could be used as an objective tool for UDS interpretation (12). The urodynamic scores were as follows: MCC, BC, detrusor activity, and detrusor-sphincter synergy were graded and summed up to obtain UDS score. MCC, BC, detrusor activity, detrusor-sphincter synergy quantified values >1, and UDS score >4 were abnormal (13).

### LUTS questionnaire

Patients were quantitatively appraised for LUTS according to the Lower Urinary Tract Dysfunction Research Network Symptom Index-29 (LURN SI-29) (14). The LURN SI-29 calculates a total score based primarily on five brief scales measuring urgency, incontinence, voiding difficulty, nocturia, and pain, plus nine separate questions about frequency, nocturnal urgency, sensation, incomplete emptying, dribbling after urination, bifurcation of urine flow, and overall distress. Total scores were positively correlated with LUTS and higher scores mean worse symptoms. The reliability of the scale has been verified and could provide granular and precise information about LUTS (15). In the study, the children and parents completed questionnaires based on the patients’ LUTS symptoms with the help of a professional medical practitioner. In an initial examination of the reliability of the LURN SI-29, all scales had internal consistency (Cronbach's alpha) greater than 0.7 (15-17).

### Strengths and Difficulties Questionnaire (SDQ)

The questionnaire was completed by the children's families under the guidance of medical professionals. SDQ comprises 25 items distributed into five scales. Emotional problems, conduct problems, hyperactivity, and peer interaction problems, with higher scores considered abnormal, were added together to obtain a total difficulty score; pro-social behavior, with lower scores considered abnormal. SDQ has been extensively evaluated and widely applied to assess behavior disorders. The reliability and validity of SDQ make it a friendly screening measure of psychosocial problems for children and adolescents (18-20). The Cronbach's alpha for this questionnaire was 0.784 (18, 21, 22). The threshold values of abnormality for each factor were total difficulty score ≥ 17, emotional problems ≥ 5, conduct problems ≥ 4, peer interaction problems ≥ 4, hyperactivity ≥ 7, and pro-social behavior ≤ 4; the normal level was defined as total difficulty score ≤ 13, emotional problems ≤ 3, conduct problems ≤ 2, hyperactivity ≤ 5, peer interaction problems ≤ 2, and pro-social behavior ≥ 6; the middle range was the borderline status. In administering the SDQ questionnaire to patients, emotional problems, conduct problems, hyperactivity, peer interaction problems, pro-social behavior, and total difficulty scores of the questionnaire were categorized into normal and abnormal groups according to their scores as required, with the former comprising both normal and borderline scores. The questionnaire was completed by the children's families under the guidance of medical professionals.

### Group processing

Children with primary TCS were divided into two groups according to the preoperative UDS parameters: The normal bladder function group (Normal group) and the bladder dysfunction group (Abnormal group). The postoperative urinary system of the children in the two groups were analyzed according to the alterations of the postoperative UDS parameters. Normal bladder function was defined as UDS score ≤4, and improved bladder dysfunction was described as a decreased UDS score (13). According to preoperative symptoms and postoperative clinical outcomes, lower extremity pain, motor dysfunction, and intestinal tract dysfunction were classified as improvement (symptoms improved), stability (no change in symptoms but no progression), ineffectiveness (failure to control the disease and continued progression), and aggravation (symptoms worsened), with improvement and stability as effective treatment and ineffectiveness and aggravation as ineffective treatment (23).

### Statistical Analysis

SPSS 22.0 software was used for statistical analysis of the data. The differences of each parameter of UDS and each index of LURN SI-29 were tested for normality at P < 0.05 and conformed to non-normally distribution, expressed as median and quartiles [M (P25, P75)], with Wilcoxon matched-pairs signed rank test. Each entry of the SDQ questionnaire before and after surgery in children with primary TCS was tested with McNemar's test. Count information of Normal and Abnormal groups was expressed as frequency and rate (%), and comparisons were made using the Pearson chi-square test. Two-sided test, test level α=0.05, P <0.05 was considered statistically significant.

## RESULTS

### Baseline information

A total of 66 children fulfilled the criteria, comprising 35 male cases and 31 female cases (Table-1 and Table-S2). Age at operation ranged from 71 to 120 months, with a mean age of 89.5 ± 13.7 months. Follow-up time ranged from 3 to 10 months, with a mean follow-up duration of 4.6 ± 2.0 months. Cutaneous stigmata such as hyperpigmentation and hypertrichosis were present in 52 patients (78.8%). 35 (53.0%) patients presented with preoperative lower extremity disorders, and 19 (28.8%) patients presented with preoperative intestinal tract dysfunction. There were 44 patients with preoperative bladder dysfunction, including 7 patients (15.9%) with constipation and 9 patients (20.5%) with fecal incontinence.

**Table 1:** Characteristics of patients undergoing detethering surgery for tethered cord syndrome.

TCS Type		Sacral Cutaneous Findings		Lower Extremity Dysfunction		Intestinal Tract Dysfunction		Bladder Dysfunction	
Type	n (%)	Symptom	n (%)	Symptom	n (%)	Symptom		Symptom	n (%)
Transitional LMMC	33 (50.0)	Hyperpigmentation	29 (43.9)	Normal	31 (47.0)	Normal	47 (71.2)	Normal	22 (33.3)
Dorsal LMMC	14 (21.2)	Normal	14 (21.2)	Weakness of both feet	20 (30.3)	Constipation	11 (16.7)	Urinary incontinence	20 (30.3)
Filum terminale lipoma	11 (16.7)	Tufts of hair	14 (21.2)	Clubfoot	11 (16.7)	Fecal incontinence	9 (13.6)	Weak stream	11 (16.7)
Caudal LMMC	9 (13.6)	Sacral mass	14 (21.2)	Ankle deformity	2 (3.0)			Enuresis	11 (16.7)
		Dimple	10 (15.2)	Plantar flexion weakness	2 (3.0)			Dysuria	9 (13.6)
		Skin tag	5 (7.6)	Left leg weakness	2 (3.0)			Urgency	8 (12.1)
								Urine hesitation	2 (3.0)

LMMC = lipomyelomeningocele

### Comparison of UDS pre- and post detethering

Preoperatively, a considerable proportion of cases presented with aberrant MCC (51.5%), BC (65.1%), detrusor activity (81.8%), detrusor-sphincter synergy (63.6%), and UDS scores (60.6%). Postoperatively, a larger number of cases exhibited abnormal MCC (60.6%), BC (72.7%), detrusor activity (90.9%), detrusor-sphincter synergy (77.3%), and UDS scores (75.8%). The patients’ UDS scores before and after surgery were: improved (21, 31.^8^%), unchanged (14, 21.^2^%), and worsened (31, 47.0%). The urodynamic parameters, including Qmax, PVR, MCC, BC, and MCC (% of CV norm), showed no statistically significant differences before and after detethering. The UDS scores, including MCC, BC, and detrusor activity, showed no statistically significant differences before and after detethering. However, significant differences were observed in bladder detrusor sphincter synergy and urodynamic score (Table-2).

**Table 2:** The UDS, LUTS and SDQ results before and after tethered cord syndrome.

	Variables	Preoperative M (P25, P75)	Postoperative M (P25, P75)	Z / X^2^	P
Urodynamic testing	Qmax	12 (8.75, 15.00)	12.00 (5.75, 15.00)	-0.584	0.559
	PVR	20 (6.75, 50.00)	20.00 (5.00, 41.25)	-0.309	0.758
	MCC	221.00 (190.25, 2540.00)	221.00 (180.25, 259.00)	-0.348	0.728
	BC	23.00 (17.75, 25.00)	21.00 (16.75, 25.00)	-1.114	0.265
	MCC (% of CV norm)a	0.95 (0.79, 1.06)	0.89 (0.72, 1.03)	-1.818	0.069
UDS score	MCC	1.00 (0.00, 2.00)	1.00 (0.00, 2.00)	-1.009	0.313
	BC	1.00 (0.00, 2.00)	1.00 (0.00, 2.00)	-1.315	0.189
	Detrusor activity	2.00 (1.00, 3.00)	3.00 (1.00, 4.00)	-1.860	0.063
	Detrusor sphincter synergy	1.00 (0.00, 2.00)	1.00 (1.00, 2.00)	-2.561	0.010
	Overall score	4.00 (2.00, 7.00)	6.00 (3.75, 8.00)	-2.485	0.013
Lower Urinary Tract Dysfunction Research Network Symptom Index-29	Urinary urgency	0.00 (0.00, 16.67)	4.17 (0.00, 18.75)	-2.582	0.010
	Urinary incontinence	12.50 (0.00, 31.25)	16.67 (0.00, 50.00)	-2.060	0.039
	Voiding difficulty	5.00 (0.00, 41.25)	17.50 (0.00, 51.25)	-2.679	0.007
	Nocturia	0.00 (0.00, 0.00)	2.00 (0.00, 0.00)	-0.868	0.385
	Urinary pain	0.00 (0.00, 0.00)	0.00 (0.00, 12.50)	-2.066	0.039
	Total scores	13.28 (3.80, 29.43)	17.71 (11.07, 32.90)	-3.204	0.001
Strengths and Difficulties Questionnaire	Emotional problems			0.030	0.861
	Normal n (%)	31 (47.0)	30 (45.5)		
	Abnormal n (%)	35 (53.0)	36 (54.5)		
	Behavioral problems			0.192	0.662
	Normal n (%)	54 (81.8)	52 (78.8)		
	Abnormal n (%)	12 (18.2)	14 (21.2)		
	Hyperactive attention inability			0.153	0.696
	Normal n (%)	47 (71.2)	49 (74.2)		
	Abnormal n (%)	19 (28.8)	17 (25.8)		
	Peer relationship problems			0.125	0.723
	Normal n (%)	39 (59.1)	40 (60.6)		
	Abnormal n (%)	27 (40.9)	26 (39.4)		
	Social behavior			0.000	1.000
	Normal n (%)	64 (97.0)	64 (97.0)		
	Abnormal n (%)	2 (3.0)	2 (3.0)		
	SDQ score			0.131	0.717
	Normal n (%)	42 (63.6)	42 (63.6)		
	Abnormal n (%)	24 (36.4)	24 (36.4)		

UDS = urodynamics; LUTS = lower urinary tract symptoms; SDQ = Strengths and Difficulties Questionnaire; Q_max =_ maximum flow rate; PVR = post void residual; MCC = maximum cystometric bladder capacity; BC = bladder compliance

### The variations of LUTS, hydronephrosis, and urinary tract infection pre- and post detethering

In order to further corroborate the change in LUTS following surgery, quantitative scoring of patients’ LUTS was performed and compared before and after the procedure. The results revealed that there were statistically significant differences in urinary urgency, incontinence, voiding difficulty, urinary pain, and total score. Postoperative follow-up demonstrated deteriorations in urinary incontinence, urgency, dysuria, and overall LUTS score. However, nocturia did not exhibit a statistically significant difference between preoperative and postoperative follow-up, indicating that it did not improve (Table-2). Preoperative hydronephrosis 12 patients (18.2%), postoperative reduced to 7 patients (10.6%); Preoperative urinary tract infection 8 patients (12.1%), postoperative reduced to 3 patients (4.5%).

### Psycho-behavioral changes pre- and post-detethering

Grounded on qualitative analysis of the SDQ data before and after the surgery, we determined whether the children's psychological behavior improved or deteriorated. Statistical results showed that emotional problems, character problems, hyperactivity, peer interaction problems, engaging in pro-social behavior, and total difficulty score did not altered (All P > 0.05, Table-2).

The detection rates of abnormal preoperative scores were relatively high, with emotional problems, conduct problems, hyperactivity, peer interaction problems, pro-social problems, and total SDQ scores exhibiting abnormal rates of 53.0%, 18.2%, 28.8%, 40.9.%, 3.0%, and 36.4%, respectively. The detection rates of abnormal postoperative scores were found to be 54.5%, 21.2%, 25.8%, 39.4%, 3.0%, and 36.4%, respectively.

### Group processing results

There were no statistically significant differences observed between the two groups for gender distribution, lower extremity pain and motor dysfunction, and intestinal tract dysfunction following surgery (all P > 0.05, Table-3). However, it was noteworthy that the proportion of patients exhibiting improved or stable bladder function post-surgery was higher in the Abnormal group compared to the Normal group (χ2 = 10.482, P = 0.001).

**Table 3:** Comparison of the improvement or deterioration of children with TCS in the two groups after surgery.

Variables		Total (n = 66)	Normal group (n = 36)	Abnormal group (n = 30)	X^2^	P
**Gender**					2.512	0.113
	Boy (n, %)	29 (43.9)	19 (28.8)	10 (15.2)		
	Girl (n, %)	37 (56.1)	17 (25.8)	20 (30.3)		
Lower limb sensory and motor dysfunction					0.084	0.772
Effective (n, %)		45 (68.2)	24 (36.4)	21 (31.8)		
Ineffective (n, %)		21(31.8)	12 (18.2)	9 (13.6)		
Bowel dysfunction					1.744	0.187
Effective (n, %)		43 (69.7)	26 (39.4)	17 (25.8)		
Ineffective (n, %)		23 (34.8)	10 (15.2)	13 (19.7)		
Bladder dysfunction					10.482	0.001
Effective (n, %)		34 (51.5)	12 (18.2)	22 (33.3)		
Ineffective (n, %)		32 (48.5)	24 (36.4)	8 (12.1)		

## DISCUSSION

TCS is known to be a progressive disease, and bladder dysfunction is closely related to the development of the disease. Bladder wall thickness, urinary nerve growth factor value, and UDS can be used as indicators to predict disease progression All kinds of indexes such as the bladder wall thickness, urinary nerve growth factor values, UDS, etc can be used as a prediction index of disease progression (24, 25). Among them, the UDS is the gold standard method for determining the type and degree of bladder dysfunction in children with TCS and can be an objectively useful test for indirectly determining neurological function (26). The urodynamic manifestations of children with primary TCS are bladder dysfunction during the filling phase, the voiding phase, or both, with predominantly hyperactive urethral reflexes, DSD, reduced BC, anosognosia, and hypocontractility of the detrusor (12, 27). The abnormal urodynamic findings in the 66 children in our study were similar to those described above, mainly in terms of abnormalities in MCC, BC, detrusor activity, detrusor-sphincter synergy, Qmax, and PVR, and the changes in Qmax, PVR, MCC, BC, and detrusor activity before and after the surgery were not significant. This further confirms that as primary TCS progresses it can lead to irreversible damage in bladder dysfunction.

The UDS score is obtained by summing the graded quantitative values of these four parameters, and its application complements the isolated parameters in assessing the overall picture of neurological dysfunction preoperatively or deterioration postoperatively (12). It has been shown that the combination of UDS scores and the values of the parameters reflect neurological dysfunction more comprehensively and accurately. The UDS scores are also considered to be a useful indicator of improvement or deterioration of bladder function (13). The difference between pre- and post-surgical UDS scores in children with primary TCS was statistically significant, and the quantitative scores were higher in the postoperative period than in the preoperative period. In addition, the results of the LUTS questionnaire further confirmed the deterioration of the patients’ LUTS postoperatively. By comparing the UDS scores and the LUTS questionnaire, it was easy to find that the bladder function of the patients had an overall decreasing trend after surgery. This is on the one hand due to the older age of intervention when severe nerve damage existed. At this time, prophylactic DS has been unable to reverse the injury. On the other hand, it may be due to further progression of the disease or damage to the nerves by manipulation during surgery.

DS has been controversial, with the main point of contention being the need for prophylactic surgery in asymptomatic children. The arguments for prophylactic DS are that a certain (undefined) percentage of these patients will go on to develop symptomatic primary TCS (28). Although the procedure is tempting and efficacious, a few patients do not derive any improvement in long-standing. It was reported that seven children with unremarkable preoperative UDS developed bladder dysfunction subsequent to surgery with five (71.4%) culminating in permanent postoperative bladder dysfunction (6). McVeigh and colleagues came to a comparable conclusion that DS involved noteworthy hazards of spinal cord injury and high rates of retethering (29). However, a related study of adult spinal embolism may also indirectly illustrate the presence of deterioration in bladder function over time in children with asymptomatic TCS exhibiting lower urinary tract symptoms during adulthood (30). It is undeniable that asymptomatic patients with primary TCS may experience deterioration of bladder function after surgery, but failure to operate in such patients may result in bladder dysfunction progressing to irreversible damage. Therefore, we divided 66 study subjects into normal and abnormal groups for comparative study, the improvement of bladder function in abnormal group after surgery was better than that in normal group, meanwhile, we compared gastrointestinal and lower limb symptoms, and there was no difference between normal and abnormal groups before and after surgery. Therefore, asymptomatic children should be carefully considered for surgery, and it is recommended to carry out prior conservative treatment before surgery and follow-up observation. Research on the beneficial effects of prophylactic DS in asymptomatic patients remains to be confirmed in large sample controlled, prospective studies.

In the era of individualized medicine, the clinical management of each patient should be thoughtfully considered. The ICCS recommends that the overall risk profile of DS should be carefully considered for asymptomatic patients or those with fixed, mild abnormalities (31). Surgery is only advocated if observation risks outweigh intervention. A meta-analysis on the treatment of TCS highlighted that abnormal UDS results can be used as supplementary evidence for the implementation of surgical intervention and that a more standardized treatment plan for bladder dysfunction in patients can be developed based on the UDS variations (32). In the study, 66.7% of patients with normal preoperative UDS had worsened UDS after surgery, whereas 71.4% of patients who presented abnormal preoperative UDS had improved or stabilized UDS. This illustrates that in terms of bladder function, surgical treatment is generally not recommended for children without preoperative bladder impairment, whereas the presence of abnormal preoperative UDS may be additional evidence for aggressive surgical intervention.

Children with primary TCS, as well as caregivers and medical practitioners, commonly had a multitude of concerns regarding neurogenic bladder, pharmacological management, and financial expenditure. The numerous and wide-ranging mental concerns plague patients all the time. Indeed, the pervasive and far-reaching nature of these concerns underscores the profound impact that primary TCS can have on the psychological well-being of pediatric patients and their families. It was reported that children and adolescents with lower urinary tract dysfunction appeared to be 2.6-fold more likely than normal to develop emotional and behavioral problems. Alternatively, the presence of constipation alongside bladder dysfunction further exacerbates the psychological symptoms (33). A population-based investigation showed that children with elimination disorders such as urinary and fecal incontinence were demonstrated as a nexus with anxiety and depression symptoms (34). These psychological disturbances hold a negative impact on management of urinary diseases, patient adherence to treatment, and overall clinical outcomes, thereby creating an ever-worsening cycle (35). To assess whether emotional and behavioral problems are more common in children and adolescents with primary TCS, our institution used the SDQ questionnaire to investigate whether surgery can alleviate the misery endured by patients. It was observed that children diagnosed with primary TCS exhibited significantly elevated rates of detection for all types of SDQ compared to their typically developing peers in this study. In particular, they displayed notably higher rates of emotional problems (52.5%) and peer relationship problems (39.3%), indicating a significant burden on their psychological well-being. The presence of lower limb deformities and the inability to control urination and defecation in children with primary TCS contributed to their alienation from peers or peer alienation. In turn, this could precipitate a cascade of negative psychological outcomes such as diminished self-worth and other mental health disorders. The findings indicated that the presence of primary TCS severely affected children's psychological behavioral problems. Moreover, there was no significant difference in psychological behavioral assessment pre- and post-surgery, possibly due to the incomplete resolution of symptoms, limited follow-up duration, and insufficient attention to a family environment and psychological support and education. Therefore, healthcare professionals and patients’ families should enhance psychological counseling and health education for children with primary TCS.

## CONCLUSIONS

In the era of individualized medicine, it is imperative to meticulously contemplate the multidisciplinary clinical management of each patient with primary TCS. In terms of bladder function, for children with primary TCS who present abnormal or progressive bladder dysfunction, surgery is advocated; Children with primary TCS exhibiting normal or non-progressive bladder dysfunction could be treated conservatively with close observation. Notably, children with primary TCS have severe emotional and behavioral disturbances, leading to reduced treatment adherence. Psychological counseling and health education for children with primary TCS should be strengthened to ensure optimal care and delivery of the most favorable results, both preoperatively and postoperatively.

## Data Availability

The datasets generated during and/or analyzed during the current study are available from the corresponding author on reasonable request.

## ABBREVIATIONS

TCS: = Tethered cord syndrome
LUTS: = Lower urinary tract symptoms
LMMC: = lipomyelomeningocele
UDS: = Urodynamics
ICCS: = International Children's Continence Society
Qmax: = Maximum flow rate
PVR: = Post void residual
MCC: = Maximum cystometric bladder capacity
BC: = Bladder compliance
LURN SI-29: = Lower Urinary Tract Dysfunction Research Network Symptom Index-29
SDQ: = Strengths and Difficulties Questionnaire
